# Addressing the surgical waiting list through comprehensive geriatric assessment

**DOI:** 10.1016/j.fhj.2025.100254

**Published:** 2025-05-15

**Authors:** Karina James, David Burberry, Duncan Soppitt, Elizabeth Davies, Jugdeep Dhesi

**Affiliations:** a Consultant Geriatrician, Swansea University Health Board, Morriston Hospital, Swansea, UK; b Clinical Research Fellow, Swansea University Health Board, Morriston Hospital, Swansea, UK; c President of British Geriatric Society, Perioperative Medicine for Older People Undergoing Surgery (POPS), Department of Ageing and Health, Guy’s and St Thomas’ NHS Foundation Trust, London, UK, School of Life Course and Population Sciences, Faculty of Life Sciences and Medicine, King’s College London, London, UK, Division of Surgery and Interventional Science, University College London, London, UK

**Keywords:** Surgical, CGA, Perioperative, Frailty, SDM

## Abstract

Surgical waiting lists have increased in recent years, particularly in areas of socioeconomic deprivation, with the older population disproportionately affected. This has huge implications for the healthcare economy and is also observed in litigation related to poor shared decision making (SDM).

We examined how core principles of the perioperative care for the older person undergoing surgery (POPS) model could be embedded into routine care with minimal staffing. We profiled the cholecystectomy waiting list, described the population awaiting surgery and their experience on the waiting list. Our aim was to provide SDM via a comprehensive geriatric assessment for people living with frailty.

A two-tier triage system was undertaken via post and telephone for people aged over 64. All people were discussed at a multidisciplinary meeting. If people were living with frailty, they were offered a clinic appointment.

We collected prospective data on diagnoses and interventions along with comparing methods for screening.

750 patients were on the cholecystectomy list. 256 patients were aged 65 or older. The median wait time for those aged over 65 was 101.7 weeks (range 0–273 weeks).

98 patients responded to an initial survey, many describing deterioration in health while on the waiting list. 32.8% met the criteria for frailty (CFS>5) and 44 were offered clinic appointments.

Of 256 patients, a total of 51 patients were removed from the waiting list over two levels of triage; initial phone call or clinic (either virtual or face to face)

Using a triaged approach to reviewing the waiting list, one fifth of patients aged over 65 years awaiting a cholecystectomy chose not to remain on the waiting list. Over half of these were identified through simple interventions, including those by non-clinical staff. This has very significant implications if replicated across other centres with long waiting lists for surgical interventions, especially in the older population, often the cohort at highest risk of adverse outcome. These novel approaches can address sustainability in the future and improved patient care and outcomes.

## Highlights

• Waiting lists for elective surgery have increased, with up to one in seven older patients experiencing decision regret.
• Screening for frailty on elective waiting lists can highlight people who benefit from comprehensive geriatric input.
• Implementation of geriatrician-led POPS clinics with shared decision making can reduce numbers of people undergoing surgery and decision regret.
• Selecting appropriate patients for POPS clinics can save money through reducing waiting lists, medication savings and reducing specialist referrals.
• Cost savings and increased efficiency can be seen with a limited workforce.

## Introduction

Waiting lists for elective surgery have increased in recent years, particularly in areas of socioeconomic deprivation.^1^ Surgery can benefit older people but, among those who do undergo intervention, up to one in seven people describe decisional regret.^2^ Decisional regret has implications for patients, services (due to cancellations and non-attendance) and financial costs, including litigation.^2^

Shared decision making (SDM) is the process whereby a healthcare professional provides an expert view on the treatment options that are available and appropriate to an individual and supports the person, with their own lived experience and views, to make the most appropriate treatment decision. When SDM is delivered well, people are more satisfied with their care, have a better experience, and are less likely to present in crisis to healthcare services.^4^

SDM in older people can be particularly challenging. Multimorbidity and frailty, which commonly present as people age, can change the benefit–risk ratio of a particular surgical intervention.

Holistic assessment informs SDM, ensuring that surgery is only offered to those in whom the evidence suggests benefit. This holistic assessment can be delivered through comprehensive geriatric assessment (CGA). The perioperative medicine for older people having surgery (POPS) model of care uses this methodology, demonstrating improved SDM, reduction in postoperative complications and decrease in healthcare costs.3., 4.

Indeed, studies utilising CGA and SDM in POPS services describe 15% of patients choosing alternative approaches to surgery when the benefits, risks and alternatives have been fully discussed in the context of the patient’s wider health and social care issues.^5^^,^^6^ While there has been some progress in adapting and implementing this model of care in NHS hospitals, an implementation gap remains due to oft-cited barriers of lack of suitably trained workforce and funding.^7^

Our aim was to assess how core principles of this model of care could be embedded into routine clinical services with minimal additional staffing. We aimed to profile the waiting list for cholecystectomy, describe the population awaiting surgery and their experience on the waiting list, and assess the impact of a structured SDM approach on patient-level decision to proceed with surgery.

## Methodology/solution

A quality improvement project was undertaken in Morriston Hospital, Swansea, with funding obtained via the Bevan Commission to support appointment of a clinical fellow. Ethical approval was not required and patients agreed to contact on discussion with the clinical fellow.

Morriston has 850 beds, of which approximately 300 are dedicated to surgical specialties. The waiting list for cholecystectomy for Swansea Bay University Health Board was obtained and segmented according to age (younger than or older than 65 years).

A two-step triage process was undertaken:

Step 1)

People were contacted by post and asked to complete an anonymous structured patient satisfaction survey regarding their waiting list experience to date.

Step 2)

People had a phone consultation with a single research fellow using a script covering the following topics:

• Were they aware of being on the waiting list?
• Frailty – using questions to allow a clinical frailty score (CFS) to be generated
• Holistic assessment across medical, functional, social and psychological domains to inform need for comprehensive geriatric assessment (based on CRANE (Comprehensive Risk assessment and Needs Evaluation Tool) questionnaire^8^

All cases were discussed at a virtual multidisciplinary meeting. Frailty was defined as CFS >4, electronic hospital frailty risk score >5, or frailty syndromes on the CRANE questionnaire. Interventions included continence referral, virtual ward review or clinic appointments with a consultant geriatrician. The fellow did not offer clinical advice; if there were patient queries, they were invited to clinic.

Medical diagnoses and interventions were prospectively recorded. Patient satisfaction data were collected via an anonymous electronic survey following the clinic appointment and a patient-led focus group.

We compared CFS, HFRS (hospital frailty risk score) and the CRANE questionnaire as means of screening patients.

## Outcome

A total of 750 people were on the cholecystectomy list, with 34% (n=256) ≥ 65. The median wait time for those aged over 65 was 101.7 weeks (range 0–273 weeks).

Ninety-eight people (38.2%) responded to an initial survey. 58% (*n*=57) respondents described deterioration in health since the point of being added to the waiting list; 18% (*n*=17) had new unmet care needs since being referred to the waiting list, and 69% (*n*=68) were dissatisfied with the health service since referral. 32.8% met the criteria for frailty and 44 were offered clinic appointments.

Of the 265 patients, a total of 51 patients were removed from the waiting list following a two-stage process; initial phone call or clinic (either virtual or face to face). Sixty-six were not contactable after five telephone calls (25.6 %) and were excluded from intervention data.

No preoperative advice or interventions were offered to patients who were not identified as living with frailty (N=100).

Triage 1: 36 patients of those contactable (18.9%) were removed from the list following an initial phone call by the clinical fellow, for reasons including:

• Operation performed – 17
• No longer wanted operation – 16
• Change of trust – 1
• Deceased – 1
• No data – 1

Triage 2: Clinic

28% of patients contactable were offered a virtual or face-to-face clinic appointment (N=54).

**Fig. 1:**
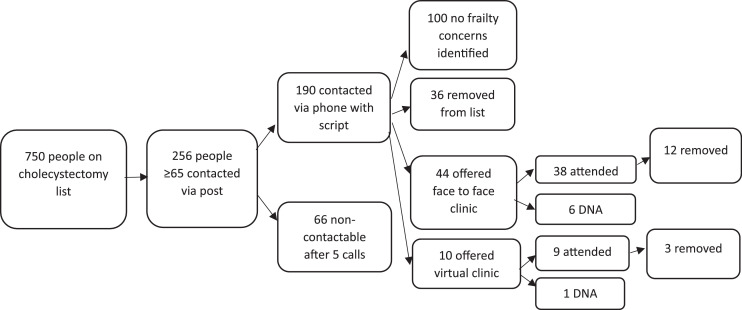
Patient outcomes.

19.9% of all respondents decided against surgical intervention (Fig. 1).

Diagnoses and therapeutic interventions made during clinic appointments are shown in the table below (Table 1).

**Table 1:** Clinic outcomes.

Intervention	Further information
Diagnostic testing (*n*=16)	Echo Holter monitor CT head Lung function test GI investigation – ERCP, sigmoidoscopy
Medication changes (*n*=6)	Medication rationalisation including stopping or titrating antihypertensives, continence management and changing bisphosphonates to denosumab. Management of newly diagnosed atrial fibrillation.
Bone health assessment with treatment (*n*=2)	Treatment commenced
Memory clinic referral (*n*=3)	All received new diagnosis of dementia
Virtual ward referral (*n*=1)	Community OT and falls assessments
Specialist referral (*n*=4)	Cardiology referral for new diagnosis of valve pathology Urology for circumcision Surgical for haemorrhoid intervention
Surgical review requested for ongoing SDM (n=4)	All went on to be removed from waiting list

## Patient experience data

**Fig. 2:**
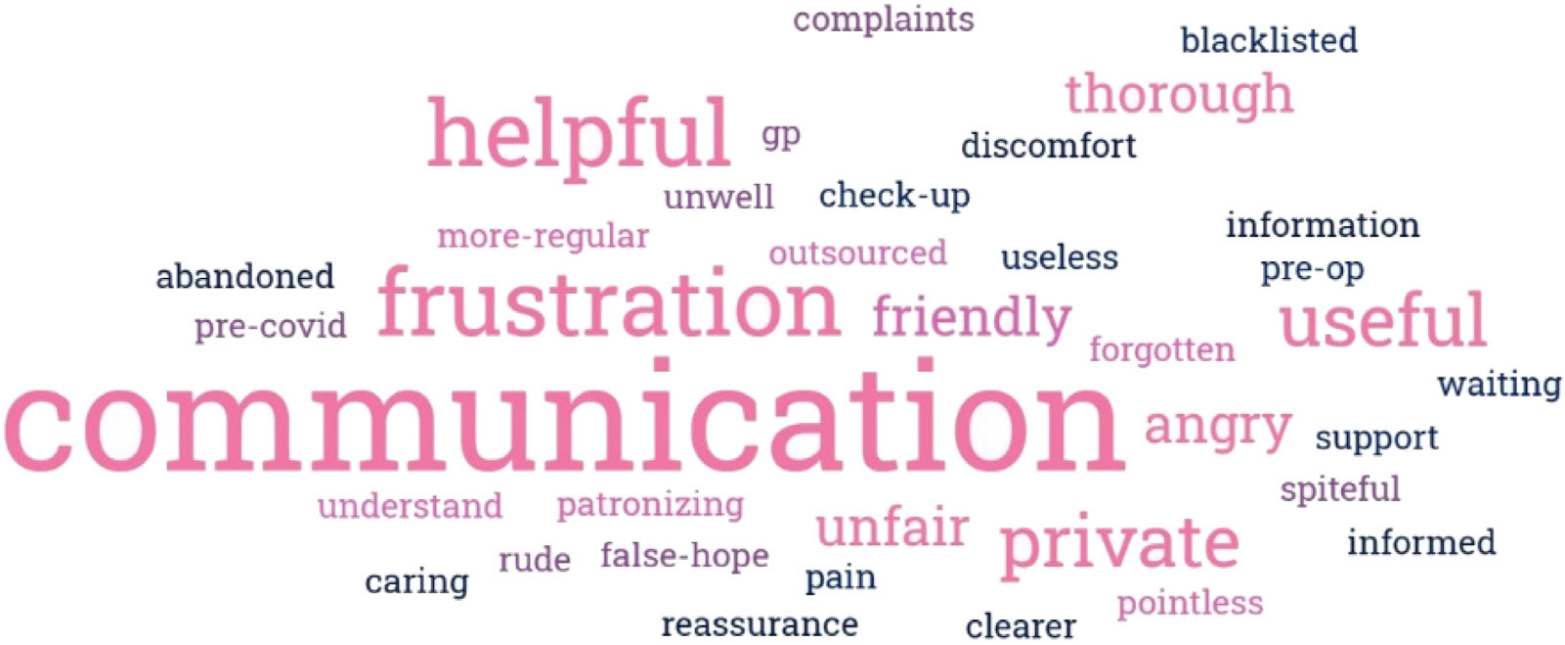
Feedback themes from focus group.

In the pre-implementation focus group, 70% of people reported dissatisfaction with their experience of waiting for surgery prior to the implementation of this service. 58% reported deteriorating health and 68% reported increased anxiety while on the waiting list. Themes expressed by the focus group prior to implementation are illustrated in Fig. 2.

People raised concerns about wasted resources, such as repeated attendance to preoperative assessment clinics despite not proceeding to surgery. They consistently described the need for streamlined communication with a clear route to a responsible clinician. This clinician could coordinate care, address concerns, and reduce duplication and waste.

Following implementation, 100% of people reported understanding of why they were attending the clinic and 100% of attending people reported a useful or very useful experience.

Feedback included ‘I could talk about things and nobody rushed me’ and ‘they helped with my general health and wellbeing, I am very grateful’.

## Conclusion

Using a triaged approach to reviewing the waiting list, one fifth of patients aged over 65 years awaiting a cholecystectomy chose not to remain on the waiting list. Of this 20%, over half were identified through simple interventions, including those by non-clinical staff. This has very significant implications if replicated across other centres with long waiting lists for surgical interventions, especially in the older population, often the cohort at highest risk of adverse outcome.

Embedding novel approaches to addressing the waiting list requires effective use of local data, developing resources to support non-clinical staff to ask the most appropriate questions (for example through using scripts) and targeting the right group of patients for interventions, using frailty as an indicator condition. Such an approach allows segmentation of the population to the right workforce, improving efficiency, reducing waste, and meeting national audit standards. With 20% of our total waiting list population choosing not to go ahead with surgery, this has significant cost saving implications. Limitations include being a single-centre quality improvement programme.

Next steps include verification of these data and replication across all general surgical waiting lists within Swansea Bay, along with collaborating with other centres to assess whether data are replicated in other sites.

## Ethics approval and consent to participate

The quality improvement project was discussed with the Swansea Health Board Ethical Committee and it was decided that ethical approval was not needed. Patients consented to discussion with the clinical fellow and attendance at clinic verbally during the telephone calls. Patient feedback at the focus group was discussed during the clinic and patients were given the opportunity to partake or decline.

## Funding

Funding was obtained via the Bevan commissions through the Planned care innovation Programme. This was to conduct a pilot study looking at characterising the cholecystectomy list and how to screen for and intervene in frail patients. There were several workshops discussing study design and data analysis, which were available to attend and it was encouraged that a written report and presentation was filed following completion of the project. Funding was to cover the salary of a junior clinical fellow for 1 year. The Bevan commission supported presentation of the data and publication, but has not aided in writing of the article itself.

## Data availability statement

The data that support the findings of this study are available on request from the corresponding author. The data are not publicly available due to privacy or ethical restrictions.

## CRediT authorship contribution statement

**Karina James:** Writing – review & editing, Visualization, Validation, Supervision, Project administration, Methodology, Investigation, Formal analysis, Conceptualization. **David Burberry:** Writing – review & editing, Methodology, Investigation, Funding acquisition, Formal analysis, Conceptualization. **Duncan Soppitt:** Writing – original draft, Investigation, Data curation. **Elizabeth Davies:** Writing – original draft, Investigation. **Jugdeep Dhesi:** Writing – review & editing, Conceptualization.

## Declaration of competing interest

The authors declare the following financial interests/personal relationships which may be considered as potential competing interests: Duncan Soppitt reports financial support was provided by Bevan Commission. If there are other authors, they declare that they have no known competing financial interests or personal relationships that could have appeared to influence the work reported in this paper.
